# Retinal hemorrhages and damages from tractional forces associated with infantile abusive head trauma evaluated by wide-field fundus photography

**DOI:** 10.1038/s41598-024-54664-y

**Published:** 2024-03-04

**Authors:** Noriyuki Azuma, Tomoyo Yoshida, Tadashi Yokoi, Sachiko Nishina, Satoko Uematsu, Mikiko Miyasaka

**Affiliations:** 1 Division of Ophthalmology, National Center for Child Health and Development, Tokyo, Japan; 2 Division of Emergency and Transport Services, National Center for Child Health and Development, Tokyo, Japan; 3 Department of Radiology, National Center for Child Health and Development, Tokyo, Japan

**Keywords:** Diseases, Medical research, Pathogenesis, Signs and symptoms

## Abstract

We evaluated the distribution and types of retinal hemorrhages (RHs) and other damages in eyes with abusive head trauma (AHT). This retrospective, consecutive case series of AHT and non-AHT conditions involved 54 children with AHT, 43 children with head bruises, and 49 children with blunt eye trauma, each of non-AHT supported by reliable witness accounts. RHs and other damage were evaluated using ophthalmoscopy and wide-field fundus photography. A variety of RH types and other damage were identified in the AHT group but not in the non-AHT group. RHs in AHT extended from the posterior pole to the far periphery in 77% of eyes and on/near the veins in 86% and arteries in 85%, most of which were in the far periphery. Retinoschisis, white-dot lesions, and retinal folds were seen even in the far periphery. RHs on/near the veins and arteries, retinoschisis, and retinal folds suggest a traumatic mechanism of the tractional force of the vitreous that is attached to the entire retinal surface. Identifying the distribution and arterio and venous origins of RHs is a key factor in determining the association with trauma. Thus, wide-field fundus photography is useful to record and evaluate the origin of the RHs and other retinal damage.

## Introduction

Abusive head trauma (AHT) is a significant problem that threatens not only vision but also survival in children. AHT occurs as a result of shaking babies and young children and sometimes from the head hitting a hard object. Retinal hemorrhages (RHs) are important signs in the eyes of infants and children with AHT, together with other signs, including intracranial hemorrhage and brain edema^1–3^. Fundus examinations often identify various types of RHs together with multi-layered RHs, vitreous hemorrhages, retinoschisis, and retinal folds throughout the entire retina^4–6^. Thus, RHs together with lethargy, irritability, enlarging head circumference, and failure to thrive in infants and young children should raise suspicion of possible AHT.

RHs also occur in infants and young children in association with basic diseases in the eye and/or whole body, and trauma^7^, in which the RHs emerge from both the arteries and veins. In contrast, accidental RHs due to venous stasis, associated with increased intracranial pressure^8–11^, chest or neck compression^12–15^, and venous sinus thrombosis^16^, emanate only from veins. Therefore, RHs arising from the healthy arteries in infants and young children occur only as a result of trauma^17–19^. Thus, identifying the sites of the RHs in both the arteries and veins may be a key way to determine the association with trauma.

It is difficult to distinguish between arteries and veins as the origins of the RHs both when vitreous hemorrhages cover the retina and when there are too many RH spots to count in the posterior retina, that is, the point at which the branches of the arteries and veins, are densely intertwined. In contrast, the branches of the arteries and veins in the retinal periphery, which separate from each other to course through those networks, are easily distinguished. The wide-field fundus camera, which has a 130-degree field of view (FOV) compared with a 30- to 45-degree FOV in traditional fundus cameras, precisely records and evaluates lesions in the retinal periphery in pediatric eye diseases, including retinopathy of prematurity (ROP) and familial exudative vitreoretinopathy (FEVR)^20–22^. However, an ultra-wide-field fundus camera with a chin rest, which has a 200-degree FOV, cannot be used for infants and young children, because their chins cannot remain stably on the rest. Contact optics as in the RetCam camera (Clarity Medical Systems, Pleasanton, CA, USA) is suitable for infants and young children.

Thus, using a wide-field fundus camera, we investigated the distribution of RH sites on/near the retinal arteries and veins in children with AHT.

## Results

### Patients

The current retrospective study included infants and young children who underwent eye and brain examinations between July 2004 and April 2020. Fifty-four children diagnosed with AHT by a child protection team (CPT) (18 girls, 36 boys; age range, 1–26 months; mean, 7.5 months) were included. In the comparison with patients with AHT, we evaluated the ocular findings in 43 patients (16 girls, 27 boys; age range, 0–28 months; mean, 6.5 months) with head bruises from accidental head trauma resulting from falling from a height of 1 m or higher or traffic accidents, and 49 patients (20 girls, 29 boys; age range, 0–30 months; mean, 16.4 months) with blunt eye trauma associated with/without facial/forehead bruises. All of non-AHT cases were supported by the reliable accounts of several witnesses. Accidental head injuries resulted from falling while attempting to grab a table, stairs, or playsets in 42 patients (98%) and a traffic accident in one patient (2%).

### Clinical features at presentation

The signs and symptoms at first admission differed markedly in children with AHT versus age-matched children with head bruises or blunt eye trauma (Table 1, Table S1). Subjects with AHT were unconscious or presented with emesis, seizures, respiratory or cardiac difficulties, skin bruising, bulging fontanelle, or pupillary abnormalities. In contrast, subjects with head bruises and blunt eye trauma were typically alert and oriented, suggesting differences in the severity of the head and ocular injuries between AHT and non-AHT conditions.

**Table 1:** Clinical features at presentation of patients.

Sign or symptom	AHT (n = 54) (%) [*p* values]	Non-AHT	
		Head bruises (n = 43) (%)	Blunt eye trauma with/without facial and forehead bruises (n = 49) (%)
Unconscious	32 (59) [0.00007; 0]	8 (19)	2 (4)
Alert	10 (19) [0; 0]	35 (81)	47 (96)
Poor sucking	6 (11) [0.03; 0.03]	0 (0)	0 (0)
Weak crying	1 (2) [1; 1]	0 (0)	0 (0)
Facial pallor	11 (20) [0.001; 0.0006]	0 (0)	0 (0)
Vomiting	11 (20) [0.16; 0.02]	4 (8)	2 (2)
Convulsions	11 (20) [0.001; 0.0006]	0 (0)	0 (0)
Opisthotonus	2 (4) [0.50; 0.27]	0 (0)	0 (0)
Tetraplegia	1 (2) [1; 1]	0 (0)	0 (0)
Respiratory disorder	7 (13) [0.02; 0.01]	0 (0)	0 (0)
Cardiac arrest	3 (6) [0.25; 0.24]	0 (0)	0 (0)
Subcutaneous hematoma	7 (13) [0.02; 0.01]	0 (0)	0 (0)
Bruises	1 (2) [1; 1]	0 (0)	0 (0)
Bulging anterior fontanelle	5 (9) [0.06; 0.06]	0 (0)	0 (0)
Pupillary abnormality	3 (6) [0.25; 0.24]	0 (0)	0 (0)

*P* values were obtained using Fisher's exact test, and presented as [*p* value between AHT and head bruises; *p* value between AHT and blunt eye trauma].

### CT and MRI findings

Head computed tomography (CT) and/or magnetic resonance imaging (MRI) scans were performed in all patients with AHT. An initial head CT was performed at the first visit in all cases. A head MRI was performed from 0 to 14 days after admission in 34 cases. CT and MRI identified a variety of intracranial lesions in all cases (Table 2, Table S1). Intracranial hemorrhages including epidural, subdural, subarachnoid and brain parenchymal hematomas, and subdural hygromas, were common. CT also identified brain parenchymal lesions, including diffuse brain edema, brain parenchymal damage, and hypoxic ischemic encephalopathy, some of which MRI later proved to be brain parenchymal injuries. Skull fractures suggested that the patients’ head had been smashed.

**Table 2:** Initial computed tomography and magnetic resonance imaging.

Intracranial lesions	AHT		Non-AHT	
			Head bruises	Blunt eye trauma with/without facial and forehead bruises
	CT (n = 54) (%) [*p* values]	MRI (n = 34) (%)	CT (n = 43) (%)	CT (n = 20) (%)
Intracranial hemorrhage	52 (96) [0; 0]	34 (100)	0 (0)	0 (0)
Subdural hematoma	45 (83) [0; 0.000001]	30 (88)	9 (21)	3 (15)
Subarachnoid hematoma	1 (2) [0.01: 1]	0 (0)	8 (19)	0 (0)
Epidural hematoma	2 (4) [0.40; 1]	0 (0)	4 (9)	0 (0)
Subdural hygroma	15 (28) [8e-5; 0.007]	10 (29)	0 (0)	0 (0)
Brain parenchyma hematoma	2 (4) [0.50; 1]	2 (6)	0 (0)	0 (0)
Brain parenchyma abnormalities	29 (54) [0; 0.00007]	14 (41)	0 (0)	0 (0)
Diffuse brain edema	26 (48) [0; 0.00005]	0 (0)	0 (0)	0 (0)
Brain parenchyma damage	5 (9) [0.06; 0.31]	14 (41)	0 (0)	0 (0)
Hypoxic ischemic encephalopathy	2 (4) [0.50; 1]	0 (0)	0 (0)	0 (0)
Bone fracture	8 (15) [0.008; 0.099]	0 (0)	0 (0)	0 (0)
Skull fracture	8 (15) [0.00015; 0.32]	0 (0)	22 (51)	5 (25)
Orbital fracture	0 (0) [1; 0.0000003]	0 (0)	0 (0)	10 (50)
No specific	0 (0) [0.04; 0.004]	0 (0)	4 (9)	4 (20)

CT was performed in 20 of 49 patients who had severe facial and head bruises together with blunt eye trauma.

*P* values were obtained using Fisher's exact test, and presented as [*p* value between AHT and head bruises; *p* value between AHT and blunt eye trauma].

*CT* computed tomography, *MRI* magnetic resonance imaging.

An initial head CT was performed at the first visit in all 43 cases with head bruises and in 20 of 49 cases with blunt eye trauma, which were associated with severe facial and forehead bruising suspected orbital bone fractures. Abnormalities were seen in 39 cases (91%) with head bruises, but there were fewer intracranial hemorrhages and more skull fractures than those with AHT. Brain parenchymal abnormalities were not identified (Table 2). Abnormalities were seen in 16 cases (80%) with blunt eye trauma with severe head bruises, but markedly fewer intracranial hemorrhages and specific orbital bone fractures were seen (Table 2).

### Lid and anterior segment ocular abnormalities

Eye examinations were performed in 108 eyes of 54 cases with AHT, 86 eyes of 43 cases with head bruises, and 53 affected eyes of 49 cases with blunt eye trauma (23 right eyes, 22 left eyes, and 8 both eyes) within 0–2 days of admission. Slit-lamp biomicroscopy did not identify any abnormalities of the lids and anterior segments in eyes with AHT or in cases with head bruises. The following were seen in eyes with blunt eye trauma: lid lacerations in five eyes (9%), subcutaneous hemorrhages/lid swelling in 19 eyes (36%), conjunctival hemorrhages in eight eyes (15%), and corneal epithelial abrasions in three eyes (6%). Cataract was not identified in any eye of each group.

### Posterior segment ocular abnormalities

**Figure 1:**
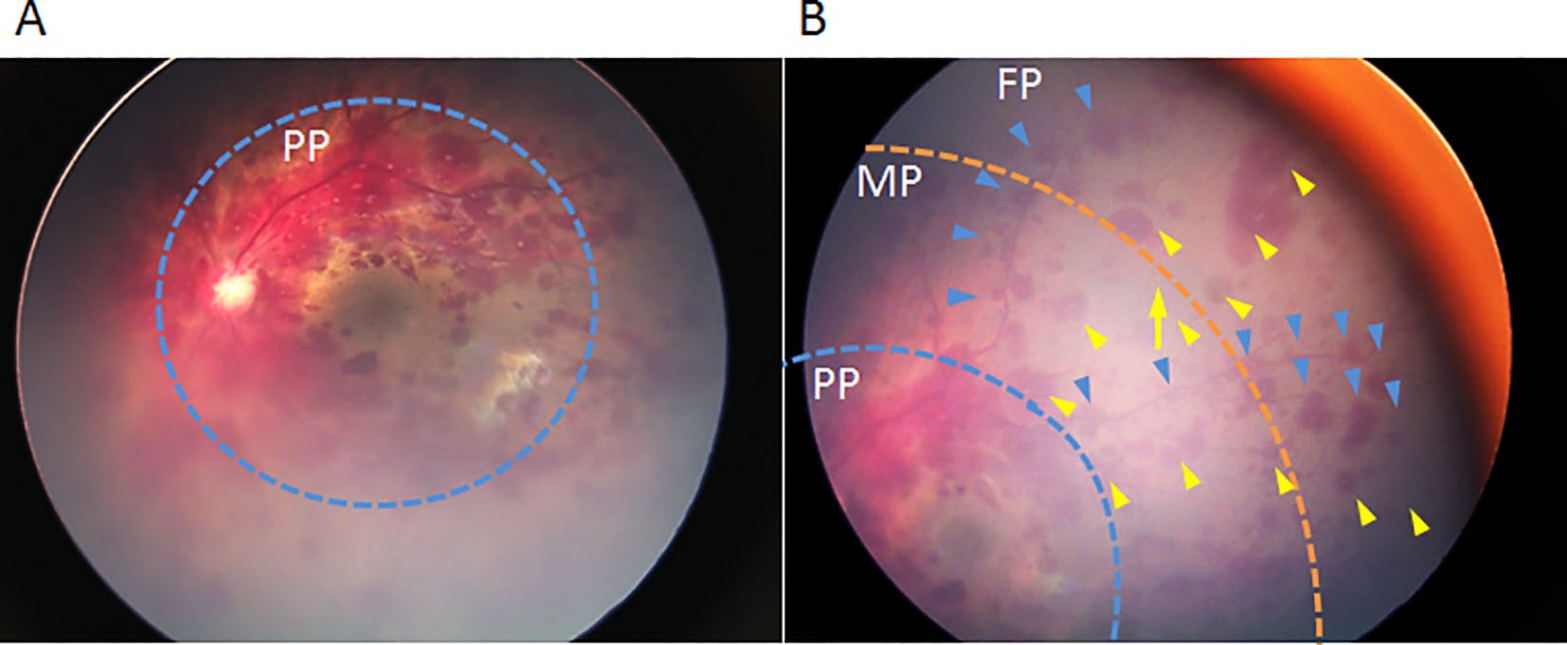
Fundus areas in fundus photographs taken by a wide-field fundus camera. Case 16 (Table S1) is that of the left eye of a 2-month-old infant. Fundus photographs were taken by a wide-field fundus camera. (**A**) The area shows largely the posterior pole (PP) (blue dotted line). (**B**) The midperiphery (MP) and far periphery (FP) of mostly one fundus quadrant. The blue dotted line indicates the border of the PP and MP; the orange dotted line, the border of the MP and FP; the arrow, an ampulla of the vortex vein; blue arrowheads, retinal hemorrhage spots on/near the branches of the vein; yellow arrowheads, retinal hemorrhagic spots on/near the branches of the artery.

Ophthalmoscopy and wide-field fundus photography were performed within 0–2 days of admission. The affected fundus areas were classified into the posterior pole (PP), midperiphery (MP), and far periphery (FP) in accordance with recommendations from the International Widefield Imaging Study Group^23,24^. The types and distributions of the RH spots in each retinal area, i.e., preretinal/vitreous hemorrhages, multi-layered RHs, and other fundus lesions, including retinoschisis, white spots on the RHs, retinal folds, and choroidal hemorrhages, were evaluated. The numbers of RH spots in the PP and the average of those in at least two quadrants of the MP and FP were counted (Fig. 1, Table S1).

In patients with AHT, ophthalmoscopy identified RHs and other lesions in 100 eyes and nothing specific in eight eyes of eight cases. In contrast, ophthalmoscopy identified no RHs or abnormalities in eyes with head bruises, and commotio retinae in one eye (2%) with blunt eye trauma. Thus, each of the RH findings described below was statistically significant between AHT and non-AHT conditions (*p* value = 0 between AHT and head bruises, and between AHT and blunt eye trauma).

The distributions and types of RHs and other retinal abnormalities in patients with AHT were analyzed by fundus photography using the RetCam camera. An extensive vitreous hemorrhage covered the entire retina in one eye, which interfered with evaluation of the retinal condition, although ultrasonography showed a circular retinal fold and large retinoschisis. Ultimately, the types and distributions of RHs and other lesions were analyzed in 99 eyes (Table 3, Table S1).

**Table 3:** Retinal hemorrhage and other fundus lesions.

Fundus lesions	n = 99 (%)
RH	99 (100)
Distribution	
PP	23 (23)
PP, MP	27 (27)
PP, MP, FP	49 (50)
RH detected on/by veins	85 (86)
Detected area	
PP	21 (25)
MP	39 (46)
FP	25 (29)
RH detected on/by arteries	84 (85)
Detected area	
PP	13 (16)
MP	29 (34)
FP	42 (50)
Local vitreous hemorrhage	30 (30)
Multilayered RH	72 (73)
Distribution	
PP	10 (14)
PP, MP	28 (39)
PP, MP, FP	34 (47)
White lesion on RH	79 (80)
Distribution	
PP	17 (22)
PP, MP	30 (38)
PP, MP, FP	32 (41)
Shape	
Dot on RH	79 (100)
Linear RH	24 (30)
Dot on transparent ILM	2 (3)
Retinoschisis	55 (56)
Distribution	
PP	27 (49)
PP, MP	25 (45)
PP, MP, FP	3 (5)
Retinal folds	12 (12)
Distribution	
PP	11 (92)
PP, FP	1 (8)
Shape	
Circular arc	12 (100)
Branch along vessels	9 (75)
Choroidal hemorrhage	15 (15)
Distribution	
PP	15 (100)

*RH* retinal hemorrhage, *PP* posterior pole, *MP* midperiphery, *FP* far periphery, *ILM* internal limiting membrane.

RHs were distributed in all four quadrants in all eyes. RHs were anteroposteriorly restricted to the PP in 23%, the PP and MP in 27%, and involved the entire retina in 50% of eyes. Preretinal and/or local vitreous hemorrhages were seen in 30% of eyes (Table 3). The numbers of RH spots in the PP and one quadrant of the MP and FP are shown in Table S1. Although the RH spots were distributed in all quadrants but anteroposteriorly in various retinal areas, many spots were on/near the branches of the retinal vessels, probably because the vitreous fibers were attached firmly to the retinal surface near the retinal vessels. We identified the origin of the RHs from veins or arteries by observing the superficial linear RH spots on/near veins or arteries in wide-field fundus photographs, because the deep and intermediate capillary plexuses differ in the arterial and venous areas^25,26^. RH spots on/near veins or arteries were undetectable in 6% of eyes, because RHs too numerous to count and/or preretinal/local vitreous hemorrhages covered many parts of the retina. RH spots on/near the veins were seen in 86% and on/near the arteries in 85% of eyes (Table 3). RH spots on/near the veins and arteries were difficult to distinguish in the PP (veins, 25%; arteries, 16%), while they were identified easily in the MP and FP (veins, 75%; arteries, 84%). Distribution pattern of RH spots was uniform in the MP and FP of all 77% of eyes, in which RHs ranged from the PP to MP and/or FP, even though arterio and venous origins were undetectable in some retinal areas. 

Other fundus lesions characteristic of AHT are shown in Table S1 and summarized in Table 3. Of the 86% of the multi-layered RHs seen in 72 eyes (73%), the lesions were distributed from the PP to the MP and FP.

**Figure 2:**
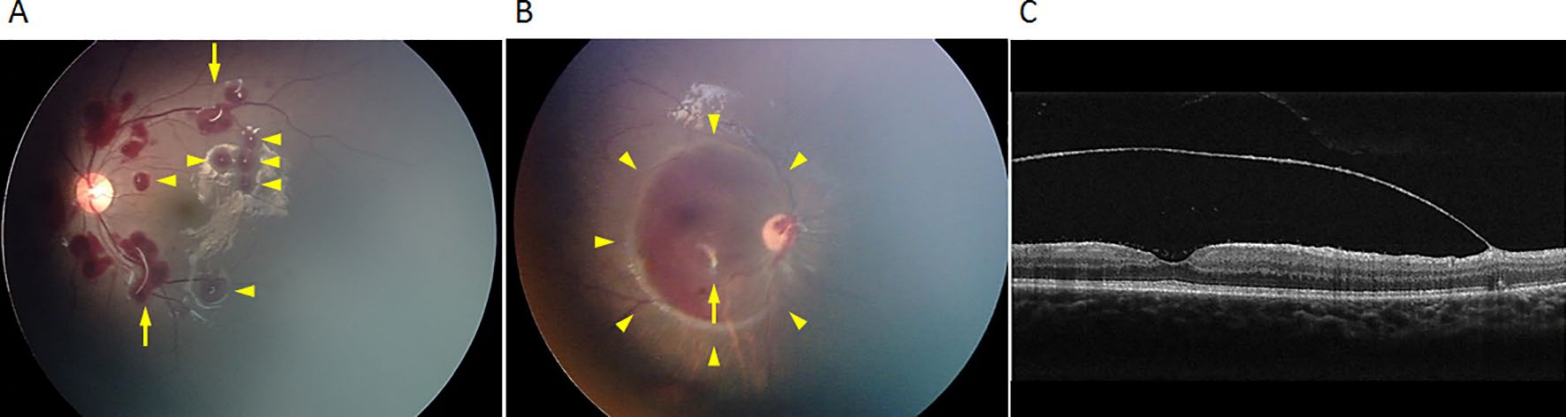
White lesions seen on the retinal hemorrhages (RHs). Case 37 (Table S1) is that of the left eye of a 20-month-old boy (**A**) and case 19 (Table S1) is that of the right eye of a 7-month-girl (**B** and **C**). White lesions are seen on the RHs. (**A**) Dot lesions (arrowheads) are on round RHs, while linear-shaped lesions are on the RHs (arrows). (**B**) A white lesion (arrow) is seen on a split transparent internal limiting membrane (ILM) (arrowheads) and on an optical coherence tomography image, in which other retinal layers are not damaged (**C**). A small RH is between the ILM and residual retina.

White-dot lesions, which are considered Roth spots, were present in the center of many RHs in 79 eyes (80%), 22% of which were restricted to the PP, 38% to the PP and MP, and 41% involved the entire retina. Although most appeared as a dot on a round RH, the shape was linear along the major axis of elliptical RHs in 30% of eyes (Fig. 2A). A white dot also was seen on the retinal surface in 49 of 55 eyes with hemorrhagic retinoschisis, two of which were on the transparent internal limiting membrane (ILM) (Fig. 2B,C).

Retinoschisis was present in 55 eyes (56%), the width of which was 3 disc diameters [DD] or more in 55% and 2 DD or less in 45%. The distribution was restricted in the PP in 49%, PP and MP in 45%, and involved the entire retina in 5%. The retinoschisis space was filled with RHs in 97%, but the transparent ILM was split with a minute amount of RHs in 3%, suggesting that splitting of the ILM occurs not by a strong influx of RH but as a result of the tractional force of the vitreous.

**Figure 3:**
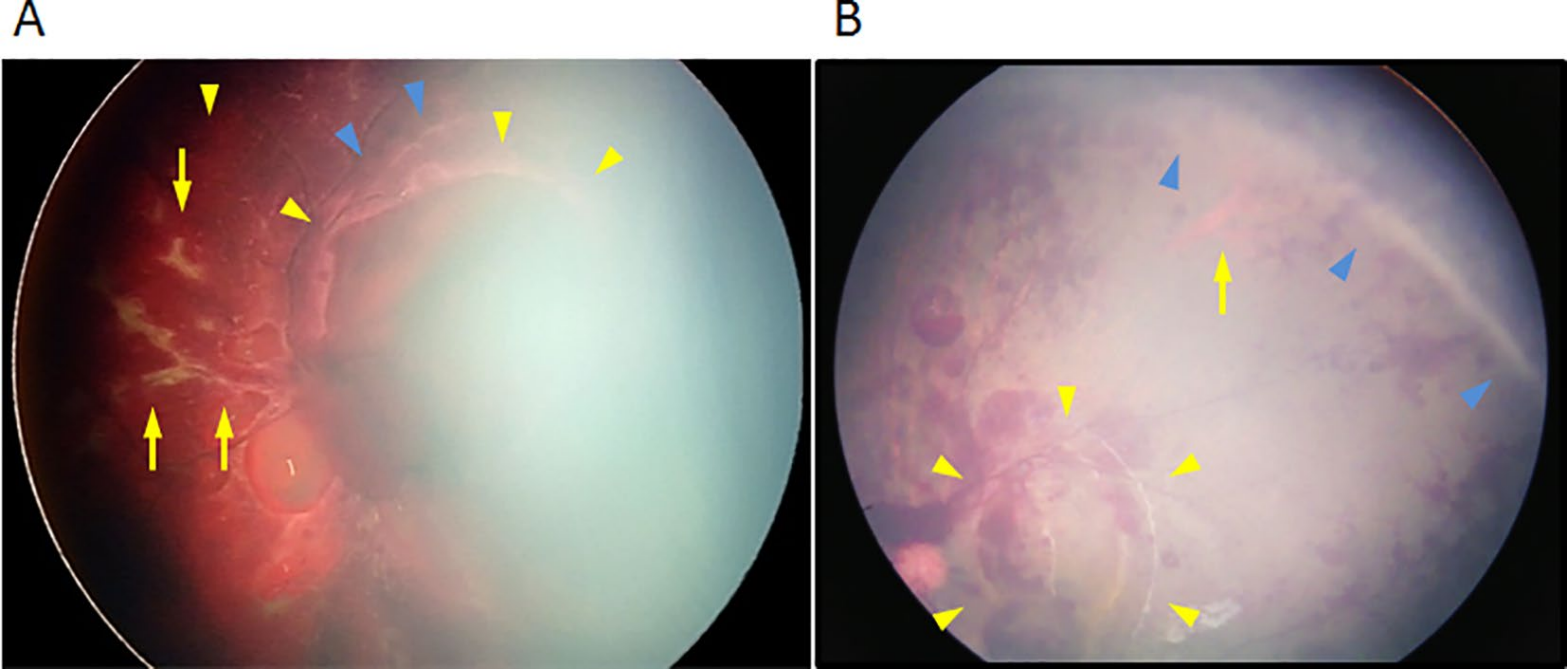
Various types of retinal folds. Case 26 (Table S1) is that of the left eye of a 5-month-old girl (**A**) and case 4 (Table S1) is that of the left eye of a 15-month-old boy (**B**). Various types of retinal folds are seen. (**A**) An arc surrounds the posterior pole (yellow arrowheads), additional folds resembling foothills (blue arrowheads), and folds along the retinal vessels (arrows) are seen. (**B**) Two arc-shaped folds are seen in the posterior pole (yellow arrowheads) and far periphery (blue arrowheads). The arrows indicate an ampulla of the vortex vein.

Retinal folds were identified in 12 eyes (12%). All appeared as arcs surrounding the disc and fovea in 80%, and a branching pattern along the retinal vessels was seen in 75% (Fig. 3A, Table 3). One arc-shaped fold was also present in the FP and extended to nearly one quadrant (Fig. 3B).

Choroidal hemorrhages were observed in 15 eyes (15%), all of which were in the PP.

The incidence and degree of RHs and other retinal abnormalities varied between each eye of an individual in some cases, that is, there was no particular findings in one eye of 8 cases and the difference was remarkable between each eye in 11 cases (Table S1).

### Clinical course

Although the RHs resolved in 4–8 months, 10 (39%) of cases with AHT required craniotomies to remove hematomas, nine required shunts for hydrocephalus, and four needed decompressive craniectomies. Thirty-three (61%) of cases were treated conservatively.

Three patients died due to cardiac and/or respiratory failures. All were in cardiac and/or respiratory arrest when admitted to our emergency department, revived, but died in 1–3 days. Among the other patients, general paralysis persisted in 12 (19%) and epilepsy in 7 (13%) cases. The diagnoses of AHT and the courses of all patients were reported to the child consultation center, Tokyo, Japan, for appropriate management: 22 cases were from our hospital and the others from the transporting hospitals.

All intracranial hematomas in patients with head bruises and blunt eye trauma were treated conservatively. All orbital fractures also were treated conservatively, because no orbital tissue incarceration or eye movement disorders were seen.

## Discussion

The present study clearly showed the differences in the occurrences of RHs between AHT and non-AHT conditions, head bruises, and blunt eye trauma. The signs or symptoms at first admission were frequent and varied in the AHT group but rare in the non-AHT group. The incidence of intracranial hemorrhages was lower and brain parenchymal abnormalities were not identified in the non-AHT group. These differences may depend on differences in the injury mechanisms between the acceleration/deceleration forces by shaking and striking forces to the head and eye. Bone fractures in AHT may result from crushing injuries in addition to shaking, although at a low incidence rate. In contrast, the incidence of bone fractures was higher in cases with head bruises, and orbital fractures often were seen in cases of blunt eye trauma. RHs, the most typical sign of AHT, were not seen in our non-AHT cases with head bruises and blunt eye trauma, even though relatively strong striking force was applied. Blunt trauma to children’s eyes results in corneal abrasion and hyphema in the anterior segment, lens damage, and macular holes, commotion retinae, RHs, retinal tears, and retinal detachments in the eye fundus^7,27,28^. A low incidence of these abnormalities in our young children without AHT may be related to less active exercise compared with older children. The soft and deformable characteristics of the ocular tissues also may tend to resist pressure forces.

The distribution and original vessels of RHs are pivotal to the diagnosis of AHT. As reported previously^4–6^, the present study confirmed that RHs with AHT were distributed throughout the retina, circumferentially and anteroposteriorly, 77% of which ranged from the PP to MP and/or FP. In adult eyes, the vascular walls of the veins and artery deteriorate with aging, which easily results in RHs, especially because of formation of varices and aneurysms. In contrast, RHs never occur in healthy retinal arteries during childhood but only in the presence of retinal and/or systemic diseases and trauma. Thus, the occurrence of RHs from the retinal artery together with the retinal vein may be a key in identifying the traumatic pathogenesis of RHs in eyes with AHT. In the current study, RHs occurred from the veins and arteries. RH spots on/near veins and/or arteries were identified in 85% of eyes. RH spots on/near the veins and artery were identified easily in the retinal periphery (MP and/or FP) where the branches of the veins and arteries separate from each other to course through those networks. Uniform distribution of RHs observed in the MP and FP is a key to determining AHT, when arterio and venous origins were not identified. If RHs occur in only the veins or arteries, the distribution pattern may not be uniform, and radial areas of RHs and those of non-RHs are alternately arranged in the retinal periphery. If RHs occur in both the veins and arteries, the distribution pattern may be uniform, with numerous RH spots covering the entire retinal periphery. Thus, a wide-field fundus camera is useful to record and evaluate distribution and original vessels of RHs in the fundus periphery.

Retinal diseases that cause RHs from the arteries and veins in children are vascular abnormalities, i.e., ROP, FEVR, hemangioma, which are easily identified by ophthalmoscopy. Systemic diseases include hematologic disorders such as leukemia and coagulation disorders; infection; inflammation; and collagen diseases, in which hemorrhages also occur systemically^7^. Among them, hematologic disorders that can cause multiple RHs in the entire retina (antero-posteriorly and 360 degrees circumferentially) in both eyes, were included in the differential diagnosis and should eliminated as a diagnosis. However, patients with AHT never have such ocular and systemic baseline diseases^1–3^.

RHs also occur as the result of a venous stasis mechanism that disturbs the blood flow in the retinal veins. Increased intracranial pressure with intracranial hemorrhage compresses the central retinal vein together with the optic nerve, known as Terson syndrome^8–11^. Chest or neck compression during cardiopulmonary resuscitation causes RHs to occur from the branches of the internal and external veins and often are associated with lid and conjunctival hemorrhages, known as Valsalva retinopathy^12–15^. Cavernous sinus thrombosis is rare but also results in intracranial hemorrhage and RHs^16^. In these venous stasis conditions, thus, RHs are present only on/near veins but not on/near arteries^8,13,16^. RHs also occur as a result of a retinal vein occlusion associated with arteriosclerosis in adults. When the central retinal vein (CRV) is partially occluded, RHs are seen on/near the main branches of the vein and surrounding capillaries, and in PP and MP, while complete occlusion of the CRV causes RHs that extend from the venous area to artery area in the entire retina, resulting in significant ischemia and retinal cell death^25,26^. However, complete cessation of blood flow in the CRV and retinal death are illogical based on the mechanisms of Terson syndrome, Valsalva retinopathy, and cavernous sinus thrombosis, which should be associated with significant occlusion of veins smaller than the CRV, upstream of the CRV, and/or branches of the upstream other than the CRV in the optic nerve, brain, and/or facial tissues.

RHs caused by trauma in children’s eyes include neonatal RHs^7^, blunt eye trauma, head bruises^17–19^, and child abuse including AHT^1–6^. However, the current study showed that RHs rarely occur following blunt eye or head trauma and frequently were 180 degrees away from the site of impact, which suggests contrecoup injury of the intraocular tissues owing to rapid movement of the vitreous, or differential tissue stiffness and viscoelastic properties^17–19^. Furthermore, the current results and those reported previously indicated that the types and distributions of RHs in eyes with AHT differed significantly from those in patients with blunt eye trauma and head bruises. RH spots resulting from trauma, which can be distributed in a wide areas of the retina, but unlikely throughout the retina, were never seen except in patients with extreme deformation of the eyeball such as that occurring with birth injury, crush of the skull and orbit, severe traffic accidents, and a fall from a great height.

Hemorrhages also occur in the intracranial space, the mechanism of which differs from that in the retina, because of differences in the tissue structures and hardness^3,29^. Presumably, the central nervous system (CNS) abnormalities result from the shifting displacement and shearing of parenchymal CNS tissues, coup and contrecoup damage of tissues. This study confirmed the high incidence of intracranial hemorrhages into the epidural, subdural, and subarachnoid spaces. Subdural hematomas were the most common. Each of these spaces has distinct cellular interfaces with bridging veins that can potentially rupture after brief translational forces generated by angular acceleration/deceleration forces.

Compared to these types of intracranial hemorrhages, RHs in eyes with AHT may occur in a mutual relationship between the vitreous and retina. RHs that occur as a result of the contrecoup force from a bruise, which impacts the vitreoretinal interface 180 degrees from the area of direct trauma, generally are characterized by a crater-like crush shape and sometimes are associated with damage to the external and anterior ocular segments^17,18,30^. In contrast, the tangential force of the vitreous, which may be produced only by shaking, is related to the characteristic tractional lesions seen on OCT images in AHT, including numerous RH spots in only the retina and choroid^31–34^. In pediatric eyes, the vitreous gel is attached firmly to the entire retinal surface, especially near the retinal vessels^35,36^; thus, numerous RH spots appear as a result of punctate traction of the vitreous fibers, i.e., figuratively speaking, pulling of the head hairs results in bleeding at many of the hair roots.

The wide-field fundus photography identified other lesions, including multi-layered RHs, white lesions on the RH, retinoschisis, and retinal folds, even in the retinal periphery, some of which have been considered to occur as a result of the tangential force of the vitreous traction^31–34^, because contrecoup damage never occurs throughout the retina as the result of one bruise^17–19^. Retinoschisis and retinal folds associated with a massive intraretinal hemorrhage have been reported in various conditions^37–39^. Such retinal folds show massive hemorrhages beneath the ILM and a double ring sign (concentric-shaped additional folds and subretinal/intraretinal hemorrhage). The nerve fiber layer under the hemorrhage is flat and unharmed due to compression of hemorrhage. In contrast, retinoschisis resulting from traction of the vitreous fibers is small and scattered. Retinal folds resulting from vitreous traction have additional radiating retinal folds/wrinkling as foothills and no massive hemorrhage. Retinal folds resulting from extreme deformation of the eyeball due to accidental crush of the skull and orbit may occur as the result of tractional force between the eyeball wall moving outward and the vitreous moving later^40,41^, the mechanism of which differs from those in AHT. In AHT, the angular velocities associated with head shaking, particularly when it terminates with direct contact and instantaneous head deceleration, are much higher than those associated with a single episode of direct blunt trauma. Furthermore, the tangential force increases along with the increasing amplitude of vitreous movement by repeated shaking. In a reported computer simulation model of the pediatric eye, the calculated stress values ranged from 3 to 16 kilo-Pascals (kPA) (1 kPa is approximately the pressure exerted by a 10 gm mass resting on a 1 cm^2^ area at the vitreoretinal interface through an episode of shaking)^42^.

Differences in the numbers of RHs and incidence of other damages between each eye of one individual may occur as the result of difference in the direction, i.e., right and left, of the head/neck rotational movement during shaking, probably related to the bodily tilt of the victims and the dominant hands of the perpetrators.

The white lesion in the center of each RH spot remains controversial. Roth spots on RHs may be fibrin in a blood clot^43^. However, the white lesions sometimes have a linear shape and those on the center of a transparent splitting ILM in the retinoschisis may not have RH clots beneath, which should be there but are not. That may suggest a structural change in the retinal surface, including a twisted ILM or a bundle of vitreous fibers, where the tractional force was applied most strongly. Additional OCT and histopathologic studies may clarify the lesions.

The limitations of the current study included the small number of patients and its non-randomized, uncontrolled, and retrospective nature. Since RHs were not seen in non-AHT cases and all cases with RHs were in the AHT group, only the characteristics of RHs in AHT cases were evaluated. Thus, the RH characteristics in AHT cases were not compared with non-AHT cases in this study.

## Conclusions

The present study showed the differences in RH occurrence in AHT and non-AHT cases due to head bruises or blunt eye trauma. RHs and other retinal damages suggest a traumatic mechanism of the tractional force of the vitreous that is attached to the entire retinal surface. Identifying the distribution and arterio- and venous origin RHs is a key for diagnosis of AHT; thus, wide-field fundus photography is useful to record and evaluate the origin of the RHs and other retinal damages throughout the retina in infant eyes during AHT.

## Methods

### Ethic declarations

The institutional review board of the National Center for Child Health and Development approved the study protocol (2020-346), which adhered to the tenets of the Declaration of Helsinki. The institutional review board granted the study an informed consent waiver and comprehensive agreement.

### Patients

The current retrospective study analyzed the clinical records of infants and young children admitted to our hospital through the emergency department who underwent eye examinations between July 2004 and April 2020. Members of a child protection team (CPT) diagnosed AHT based on the diagnostic images and ophthalmoscopic and clinical findings.

The criteria used by the CPT to diagnose AHT are serious intracranial damage and hemorrhages that do not match a scenario caused by an injury; the scenario of the associated trauma; the presence of multiple, multi-layered RHs; and no systemic baseline diseases associated with intracranial and ocular hemorrhages. Thus, no patients had systemic baseline diseases associated with intracranial and ocular hemorrhages, including the accident-related AHTs that occurred 0–2 days before admission. After analyzing all clinical findings and ruling out cases other than non-accidental head trauma, the CPT made the probable diagnoses of presumed AHT.

RHs associated with basal eye and systemic diseases were excluded from AHT, including injuries that occurred at birth, apparent accidental head injuries witnessed by multiple individuals, congenital vascular abnormalities, blood and coagulation disorders, metabolic disorders, tumors, infections, inflammation, and pathologic neovascularized retinas with ROP and FEVR.

To compare the RHs in patients with AHT, we evaluated the ocular findings in infants and children whose ages were matched with those of the patients with AHT, with a head bruise resulting from falling from a height of 1 m or higher, and those with blunt eye trauma with/without facial and forehead bruises, each of which was supported by the reliable accounts of several witnesses.

### Eye examinations

Eye examinations were performed in all patients within 0–2 days of admission. Five pediatric ophthalmologists examined the eyes of each patient using hand-held slit-lamp biomicroscope (Litz Medical, Aichi, Japan) and dilated binocular indirect ophthalmoscopy** (**HEINE Optotechnik, Gilching, Germany**)**. Two pediatric retina consultants performed ophthalmoscopy, and obtained and analyzed wide-field fundus photographs with the RetCam fundus cameras. Retina consultants identified the origin of RHs and counted the numbers of RH spots in the PP and the average of those in at least two quadrants of the MP and FP using the fundus photographs, in accordance with the recommendations regarding the retinal areas from the International Widefield Imaging Study Group^23,24^. To reduce interobserver variation in the counting of the RHs, especially in the PP that often is covered with large RHs, the numbers of dot-and-blot RHs were classified as follows: sparse (fewer than 10), moderate (10 to fewer than 50), and confluent (50 or more, or uncountable because of obscuration by large RHs or fusion of many RH spots in the PP) (Table S1). Ultrasonography (US-400 NIDEK, Gamagori, Japan) and OCT RS-3000 (NIDEK, Kamagori, Japan) also were used.

### CT and MRI examinations

Head CT imaging was performed with helical scanning on multi-slice CT (General Electric Healthcare, Milwaukee, WI, USA). In addition, head CTs were routinely reconstructed into multiplanar reformations and 3-dimensional volume rendering of the skull. MRI imaging was performed on a 3 Tesla or a 1.5 Tesla machine (Siemens, Erlangen, Germany). The MRI included T1- and T2-weighted imaging and T2 fluid attenuation inversion recovery, T2* (gradient-echo or susceptibility-weighted imaging) sequences, and diffusion-weighted sequences. A pediatric radiologist with more than 10 years’ experience reviewed all images.

### Statistical analysis

Statistical analysis was performed using Microsoft Excel statistics software in the AHT and non-AHT groups (each with cases of blunt eye trauma and head bruises). Fisher’s exact test was used to compare groups. *P* < 0.05 was considered statistically significant.

### Supplementary Information

Supplementary Table S1.

## Data Availability

The data that support the findings of this study are available from National Center for Child Health and Development but restrictions apply to the availability of these data, which were used under license for the current study, and so are not publicly available. Data are however available from the authors upon reasonable request and with permission of National Center for Child Health and Development. Either of the corresponding authors, N. A. or M. M, should be contacted if someone wants to request the data from this study.
